# Free lipid and computerized determination of adipocyte size

**DOI:** 10.1080/21623945.2018.1489335

**Published:** 2018-07-14

**Authors:** Henrik Svensson, Daniel Olausson, Agneta Holmäng, Eva Jennische, Staffan Edén, Malin Lönn

**Affiliations:** a Department of Clinical Chemistry and Transfusion Medicine, Institute of Biomedicine, Sahlgrenska Academy, University of Gothenburg, Gothenburg, Sweden; b Department of Physiology, Institute of Neuroscience and Physiology, Sahlgrenska Academy, University of Gothenburg, Gothenburg, Sweden; c Department of Medical Biochemistry and Cell Biology, Institute of Biomedicine, Sahlgrenska Academy, University of Gothenburg, Gothenburg, Sweden; d Department of Internal Medicine, Institute of Medicine, Sahlgrenska Academy, University of Gothenburg, Gothenburg, Sweden

**Keywords:** Adipocytes, cell size, lipid droplets, collagenase, microscopy

## Abstract

The size distribution of adipocytes in a suspension, after collagenase digestion of adipose tissue, can be determined by computerized image analysis. Free lipid, forming droplets, in such suspensions implicates a bias since droplets present in the images may be identified as adipocytes. This problem is not always adjusted for and some reports state that distinguishing droplets and cells is a considerable problem. In addition, if the droplets originate mainly from rupture of large adipocytes, as often described, this will also bias size analysis. We here confirm that our ordinary manual means of distinguishing droplets and adipocytes in the images ensure correct and rapid identification before exclusion of the droplets. Further, in our suspensions, prepared with focus on gentle handling of tissue and cells, we find no association between the amount of free lipid and mean adipocyte size or proportion of large adipocytes.

## Introduction

Adipocyte size is a commonly studied variable in the field of obesity and related metabolic disease.^1^ Several methods for analysis of this factor are described, all with different pros and cons.^1^ One well established technique includes collagenase digestion of adipose tissue and size determination of the adipocytes in a suspension based on direct microscopy or computerized image analysis. The presence of free lipid, forming droplets, in such suspensions is documented.^2^ The droplets implicate a bias, particularly in computerized image analysis, since they may be identified as adipocytes. This problem is not always adjusted for and some reports state that distinguishing droplets from cells is a considerable problem.^3,4^ Also, if the droplets originate mainly from rupture of large adipocytes, as often described^3-5^ without convincing original studies, this will also bias size analysis. We have previously developed^6^ and applied^7-10^ (by selection) a computerized technique, based on image analysis, to study not only average adipocyte size after collagenase digestion, but also more subtle variations in the size distribution of fat cells. Structures on the images, identified by the operator as lipid droplets, are easily excluded before the automated analysis simply by clicking on the specific structures on the computer screen.^6^ Here, adding value to the computerized technique and previous work applying the technique,^6-10^ we confirm that our ordinary manual means of distinguishing droplets and adipocytes ensure correct identification. Further, in our suspensions, prepared with focus on gentle handling of tissue and cells, we find no association between the amount of free lipid and mean adipocyte size or proportion of large adipocytes.

## Results

**Figure 1.**
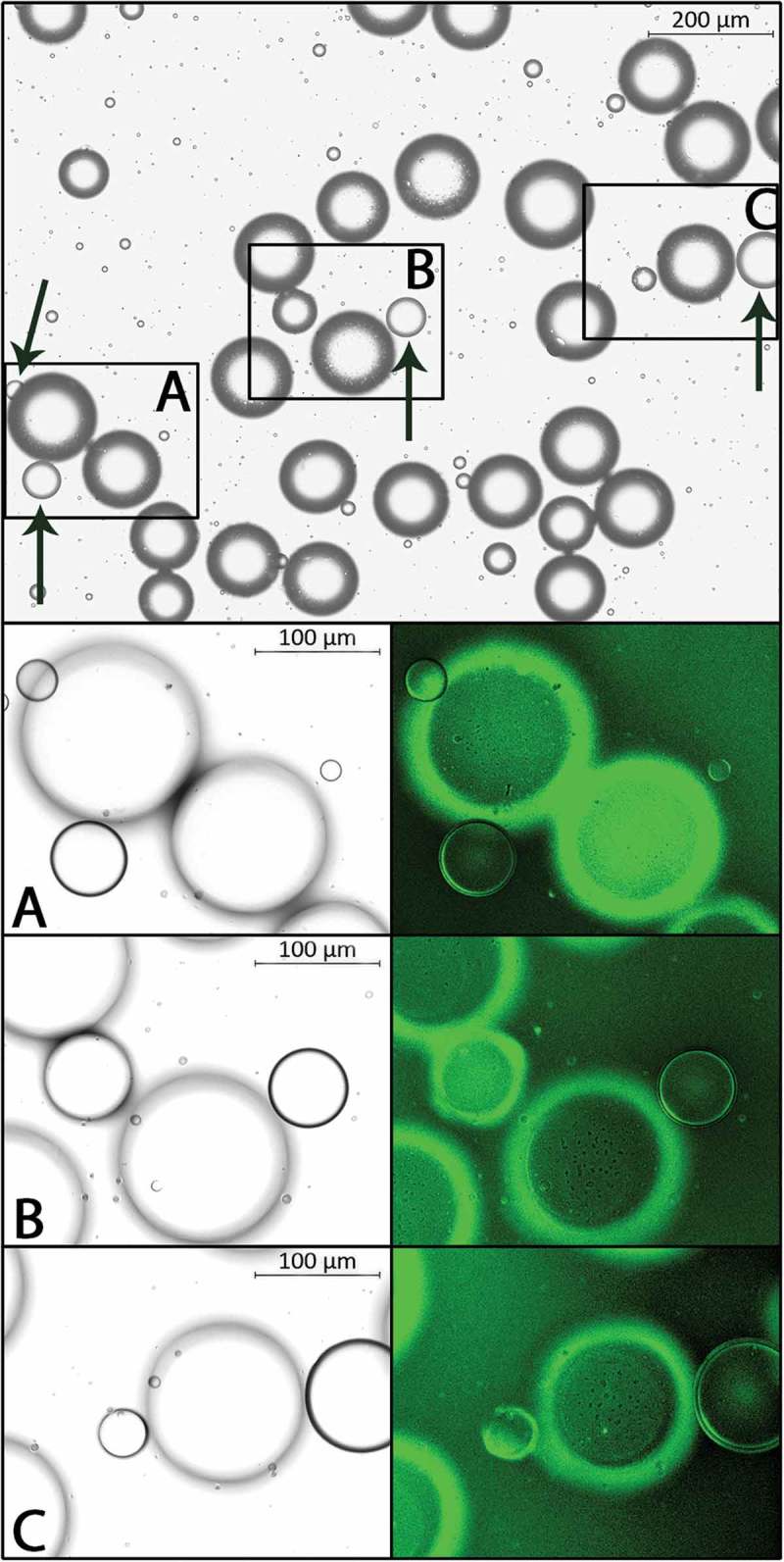
Representative images of an adipocyte suspension. Magnification 5X, bright field (top). Arrows indicate lipid droplets as identified by our ordinary manual means. Panels A-C show magnification 10X, bright field (left) and fluorescence (FITC, right); adipocyte membranes with fluorescent signal.

Addition of Cellmask Green to the suspension resulted in a distinct and homogenous staining of adipocyte membranes. Consistently, the ordinary manual means of distinguishing droplets from cells matched the definitive identification based on the absence/presence of a stained plasma membrane. Representative images of cells and droplets, with and without stain, are shown in Figure 1.

There was no difference between small and large adipocyte suspensions in total free lipid area (3,149.3 ± 1,795.6 vs 4,764.5 ± 5,399.0 µm^2^ or 0.24 ± 0.14 vs 0.37 ± 0.42% of the analyzed area, NS). The three suspensions with the lowest, and the two suspensions with the highest, amount of free lipid were all found among the suspensions with large adipocytes. There were no correlations between total free lipid area and the proportions of large (> 100 µm) and very large (> 150 µm in diameter) adipocytes, respectively (ρ = 0.103 and ρ = 0.264, n = 10, NS).

## Discussion

Direct microscopy includes the possibility of focus adjustment on every single structure to be assessed. This is a strength but also a weakness in analysis of the size of adipocytes in a suspension since the procedure is laborious and time consuming. The number of analyzed adipocytes is often limited to 100,^11^ precluding studies of size distribution curves and specific adipocyte populations. Computerized image analysis allows easily for 10-fold more assessments but some of the structures to be analyzed will inevitably be slightly out of focus. Our present results show that this is not an obstacle in distinguishing droplets and adipocytes in the images. Still, this step of the analysis requires operator input. The contour of lipid droplets versus size-matched adipocytes is generally thinner, may almost look drawn, and the droplet appears “flat”. A trained eye facilitates in this examination that is made rapidly. Identified droplets, or other non-relevant structures in the images, e. g. air bubbles, are easily excluded before analysis simply by clicking on the specific structures on the computer screen. It may be noted that the advantage of staining suspensions with a nuclear dye is limited in analysis of gray-scale images.

Collagenase digestion of adipose tissue may cause cell breakage and large adipocytes are said to be extra fragile.^3,5^ This may be due to pure physical facts and/or to membrane characteristics of the enlarged adipocyte.^12^ However, if tissue and cells are handled carefully during suspension preparation i.e. centrifugation steps are omitted, siliconized glassware are used, and if medium is supplemented with adenosine, cell breakage can be kept to a minimum. Collagenase preparations also vary in the composition of included proteases why comparisons of preparations may be valuable. With these precautions, and in keeping with a previous study,^11^ we could not identify large adipocytes as a specific source of free lipid in the suspensions.

Taken together, in analysis of adipocyte size after collagenase digestion, computerized image analysis has many advantages. The technique allows studies of size distribution curves, it is fast, and images may be stored for future reference. Lipid droplets present in the images can rapidly be identified by manual routines and excluded before analysis. Further, with gentle handling of tissue and cells we find no evidence of an association between large adipocytes and an increased amount of free lipid in the adipocyte suspensions.

## Methods

Our ordinary manual means of distinguishing between droplets and cells, based mainly on differences in the appearance of the cell/droplet contour, were evaluated using adipose tissue from an overweight woman undergoing abdominal surgery. Adipocytes were isolated essentially as described; medium with adenosine, 50 min incubation with collagenase, gentle shaking.^6^ Cellmask^TM^ Green (Life Technologies Corp, C37608), a fluorescent dye binding to the plasma membrane, was added to the cell suspension (1:1,000 vol/vol). The cells were incubated at 37 C for 5 minutes and washed twice to remove unbound stain. A chamber with the cell suspension was prepared^6^ and transferred to the microscope (Carl Zeiss, Zeiss AXIO Imager MI). Adipocytes and droplets, in four separate fields of view (in total approximately 500 assessments), were first distinguished at magnification 5X/brightfield according to the ordinary means. The assessment was performed on the screen, without adjustment of any settings, completely corresponding to the routine analysis of stored images. The definitive identification of cells and droplets was performed at 5X/fluorescence (FITC-channel) based on presence/absence of stained plasma membrane. Magnification was increased to 10X/brightfield and 10X/fluorescence for further confirmation and documentation.

The amount of free lipid in our suspensions was estimated using previously stored images included in one of our studies.^10^ Suspensions from the five women with the smallest (57.0, 58.8, 62.3, 64.4, 64.8 µm) and the largest, (mean diameter 101.7, 105.7, 109.6, 111.5, 113.5 µm) adipocytes, respectively, were selected. The center section (433,535.2 µm^2^) of three out of the twelve images of each suspension was analyzed with respect to total free lipid area (the sum of all lipid droplet cross-sectional areas; Photoshop, 1 pixel = 1.098 µm). Values are mean± SD. The Mann-Whitney U test was used for group comparisons. Spearman’s Rho was used for correlation analysis. Statistical analyzes were performed in IBM SPSS Statistics for MacOS, version 25 (IBM corp.). The studies were approved by the Ethical Review Board in Gothenburg and all subjects gave oral and written consent.
